# Diagnostic and treatment challenges of a case of primary cutaneous signet-ring cell/histiocytoid carcinoma of the eyelid

**DOI:** 10.1186/s12886-020-01685-6

**Published:** 2020-10-14

**Authors:** Mathew M. Palakkamanil, Muhammad N. Mahmood, Audrey Chan

**Affiliations:** 1 Department of Ophthalmology and Visual Sciences; Faculty of Medicine and Dentistry, University of Alberta, Edmonton, Alberta Canada; 2 Royal Alexandra Hospital, 2319 -10240 Kingsway Avenue NW, Edmonton, AB T5H 3V9 Canada; 3 Department of Laboratory Medicine and Pathology, Faculty of Medicine and Dentistry, University of Alberta, Edmonton, Alberta Canada

**Keywords:** Signet-ring, Histiocytoid, AR, GATA3

## Abstract

**Background:**

Primary cutaneous signet-ring cell/histiocytoid carcinoma of the eyelid is an extremely rare but aggressive neoplasm diagnosed primarily in elderly men. Until now there are 32 published cases of signet-ring cell carcinoma or histiocytoid carcinoma of the eyelid. We report the clinical, radiographic and histological features of the 33rd reported case of PCSRCC in the eyelid of a 73-year-old male, and review diagnostic and treatment challenges of this rare entity.

**Case presentation:**

Our case highlights a 73-year-old male who was referred for surgical correction of right eye ptosis that was present for 2 years. Upon assessment, he was noted to have an upper lateral orbital rim mass. Computed tomography (CT) noted ill-defined soft tissue thickening anterior to the right globe, predominantly pre-septal but with slight post-septal extension. The pathology revealed diffusely and deeply infiltrating tumour cells extending through the dermis, subcutis, orbicularis muscle bundles and nerve fibers; the tumour cells were noted to have a monotonous histiocytoid appearance with foamy granular eosinophilic cytoplasm. At high magnification, intracytoplasmic vacuoles and occasional intermixed signet ring cells were identified. Immunohistochemical staining revealed the tumour cells to be AE1/AE3, CK7, GCDFP-15, E-cadherin, androgen receptor stain and GATA3 positive. Final pathology report confirmed the diagnosis of primary cutaneous signet-ring cell/histiocytoid carcinoma. Further imaging failed to identify a distant primary malignancy or metastatic disease. The decision was made to attempt surgical excision of the tumor. After the bulk of the grossly apparent tumor was removed, intraoperative frozen sections were sent. Superficial biopsies of the right periorbital region were performed, which revealed extension significantly further than the gross disease. Thereafter, the patient underwent a wide orbital exenteration with reconstruction using a temporary split-thickness skin graft. Due to positive margins on final permanent sections, the patient underwent further wide resection with free muscle-skin flap reconstruction followed by adjuvant radiation treatment.

**Conclusion:**

Our case represents the 33rd case of primary signet-ring cell/histiocytoid carcinoma of the eyelid in a 73-year-old male, the first documented case with GATA3 positivity and the second documented case with androgen receptor stain positivity.

## Background

Primary cutaneous signet-ring cell/histiocytoid carcinoma of the eyelid is an extremely rare but aggressive neoplasm diagnosed primarily in elderly men. Currently, there are 32 published cases of primary cutaneous signet-ring cell carcinoma or histiocytoid carcinoma of the eyelid; the term used for diagnosis depends on the major cellular component of the lesion. The histogenesis of this tumour is controversial, however, most features point towards an apocrine differentiation [1, 2]. The diagnosis is established by the exclusion of metastases from other primary sites especially the breast, gastrointestinal tract and urinary tract. We report the clinical, radiographic and histological features of an additional case of primary cutaneous signet-ring cell/histiocytoid carcinoma in the eyelid, and review diagnostic and treatment challenges of this rare entity.

## Case presentation

Our case highlights a 73-year-old male who was referred for surgical correction of right eye ptosis that was present for 2 years. He complained that his ptosis was progressively worsening. On assessment of his driver’s license from 5 years prior, the right sided ptosis was present. His past medical history was significant for diabetes mellitus, dyslipidemia and remote head trauma resulting in a basal skull fracture. His past ocular history was significant for prior cataract extraction and primary open angle glaucoma which was controlled medically.

**Fig. 1:**
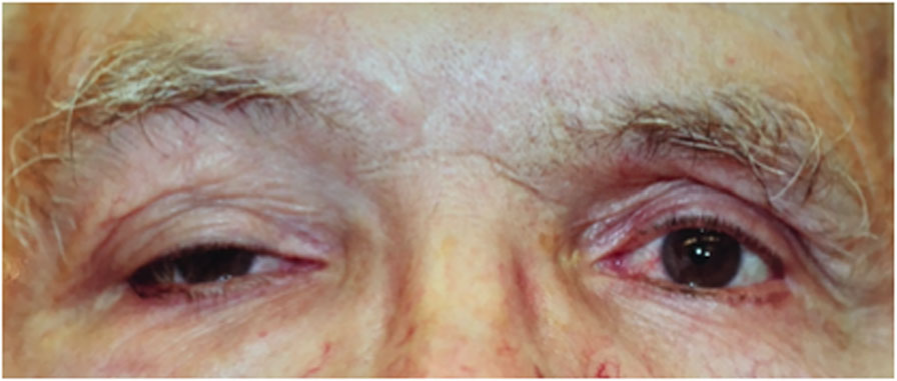
External photo showing significant right sided ptosis and apparent fullness to the superior orbital rim area

**Fig. 2:**
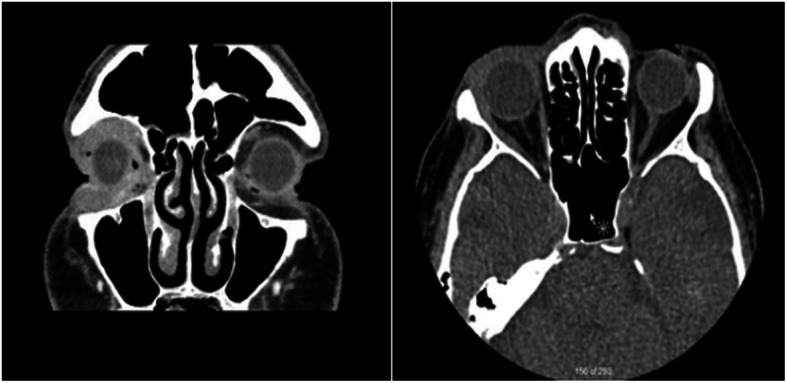
Coronal and axial contrast enhanced CT slices show an ill-defined soft tissue mass anterior to the right globe with extensive infiltration to the pre-septal and post-septal superior and inferior eyelids

On initial examination, his visual acuity was 20/25–1 and 20/20–1 in the right and left eye, respectively. His intraocular pressures were 12 mmHg bilaterally. His anterior segment examination was unremarkable. His posterior examination was unremarkable apart from glaucomatous disc damage. His ocular movements were full and there was no enophthalmos or exophthalmos. He was noted to have right-sided ptosis (Fig. 1). Upon palpation of his right orbit, he was noted to have an upper lateral orbital rim mass. The patient was otherwise asymptomatic; he did not report pain, pressure or right-sided visual deficits. Computed tomography (CT) noted ill-defined soft tissue thickening anterior to the right globe, predominantly pre-septal but with slight post-septal extension (Fig. 2).

**Fig. 3:**
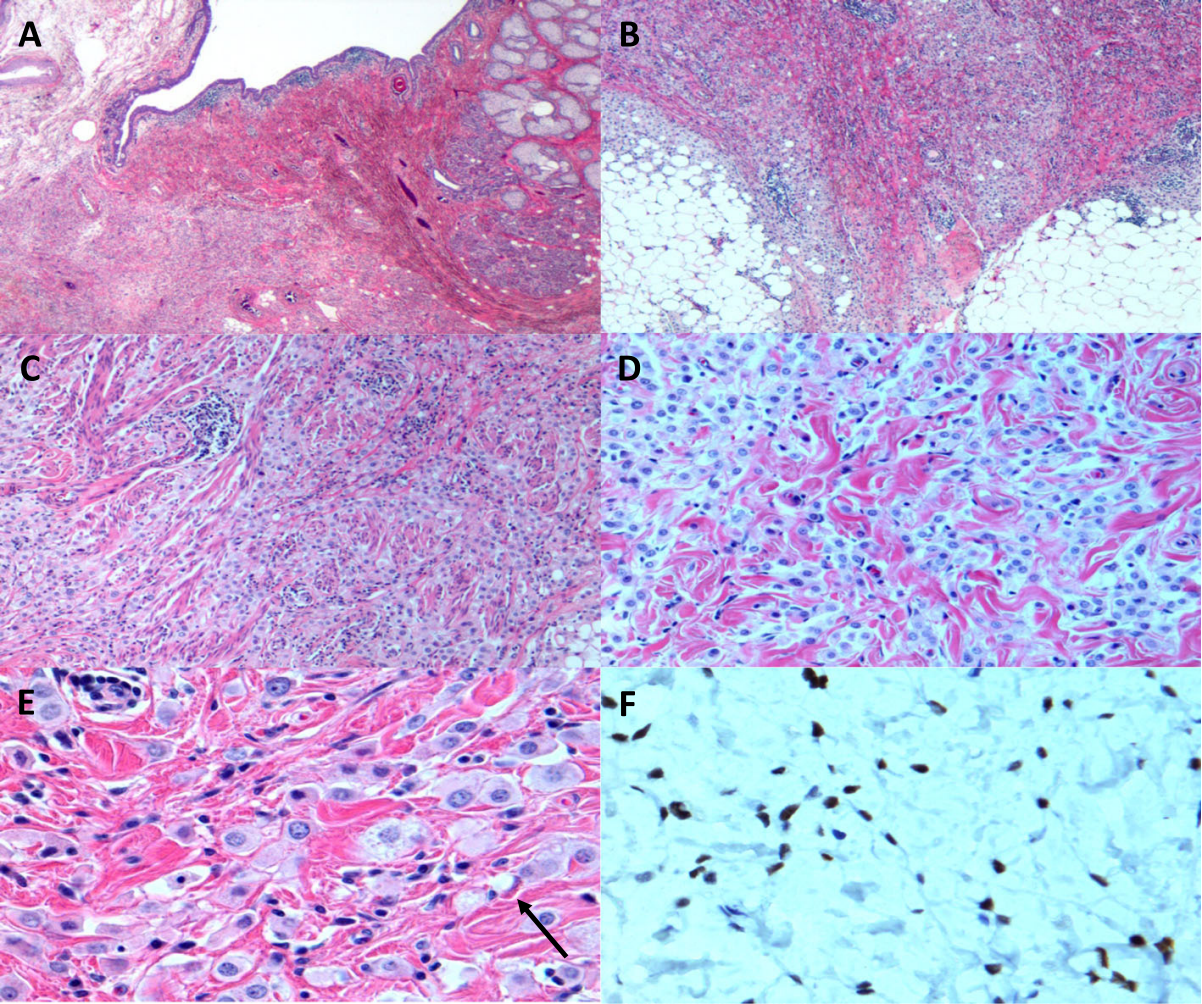
**a** 25X, H&E stained section reveals diffuse infiltration of neoplastic cells within the dermis with sparing of the epithelium. **b** 50X, H&E stained section shows infiltration of neoplastic cells into the subcutaneous adipose tissue without desmoplastic reaction. **c** 100X, H&E stained section reveals strands and aggregations of neoplastic cells interstitially arranged between collagen fibers. **d** 200X, H&E staining section shows histiocytoid cells with pale, granular eosinophilic cytoplasm with intracytoplasmic vacuolation. **e** 400X, H&E stained section shows histiocytoid morphology. The arrow points to a rare signet ring cell which is characterized by crescent-like nuclei and a main, centrally located cytoplasmic vacuole. **f** 400X, GATA3 stain shows positivity

An orbitotomy was performed for biopsy of the right orbicularis muscle and anterior orbit through an eyelid crease incision. The pathology revealed diffusely and deeply infiltrating tumour cells extending through the dermis, subcutis, orbicularis muscle bundles and nerve fibers (Fig. 3a-c); the tumour cells were noted to have a monotonous histiocytoid appearance with foamy granular eosinophilic cytoplasm (Fig. 3d). There was diffuse infiltration of the dermis with sparing of the epidermis and lack of surrounding desmoplastic reaction. In specific areas, lesional cells showed single filing. Sebaceous differentiation was not seen, and the epidermis did not show a cutaneous Paget’s disease-like change. At high magnification, intracytoplasmic vacuoles and occasional intermixed signet ring cells were identified (Fig. 3e). They were characterized by a crescent-like nuclei and a main, centrally located cytoplasmic vacuole. Rare scattered mitoses were noted. Immunohistochemical staining revealed the tumour cells to be AE1/AE3, CK7, GCDFP-15, E-cadherin, androgen receptor (AR) stain and GATA3 positive (Fig. 3f). No appreciable staining was noted with CK20, CDX2, TTF-1, S100, mammaglobin, p63, uroplakin-II, PSAP, PAX8, tryptase, CD20, CD117, CD43, WT1, calretinin and PSA stains. MIB-1 showed a low proliferative index (about 10%). Progesterone receptor stain was negative; however, estrogen receptor stain did show patchy staining (about 15–20%). Final pathology report confirmed the diagnosis of primary cutaneous signet-ring cell/histiocytoid carcinoma of the right eyelid. Systemic workup was performed with positron emission tomography (PET) and full body CT which failed to identify a distant primary malignancy or metastatic disease.

The decision was made to attempt surgical excision of the tumor. After the bulk of the grossly apparent tumor was removed, intraoperative frozen sections were sent. Unfortunately, frozen pathology was unable to differentiate tumor cells from normal inflammatory histiocytes. Thus, numerous superficial biopsies of the right periorbital soft tissue were performed, which revealed extension significantly further than the gross disease. Thereafter, the patient underwent a wide orbital exenteration with reconstruction using a temporary split-thickness skin graft. On final permanent sections, there still remained positive margins.

Later, the patient underwent further resection by the head and neck oncology team, in which further extensive resections were performed around the original rim of the exenteration site. The previous skin graft was resected, the orbital apex was further dissected, the maxillary sinus was removed and further resection into the skull base was performed. Neck dissection was performed in preparation for free muscle-skin flap reconstruction after all skin and deep margins were found to be negative.

Two rounds of adjuvant post-operative radiation were administered (5040 cGy in 28 fractions) 3 months after his reconstruction. Post-radiation PET and CT scans were negative for recurrence 6 months after his initial surgery. There was no evidence of recurrence after 4 years of follow-up.

## Discussion and conclusions

Primary cutaneous signet-ring cell/histiocytoid carcinoma of the eyelid is a rare, malignant tumour that predominantly affects elderly men with an average age of presentation of 67.1 years [3]. The typical presentation is that of gradual, painless infiltration of the eyelid. The patients often develop induration of only one eyelid. Due to this pattern of presentation it is sometimes referred to as the ‘monocle tumour’ [4]. As the most common site for this tumour is the eyelid, it is often placed in the general category of ‘site-specific adnexal neoplasm’. Rarely, primary tumours in the axilla with similar morphology have also been reported.

In regards to histopathology, primary cutaneous signet-ring cell/histiocytoid carcinoma is characterized by diffuse infiltration of the dermis by single cells, cords or rows (single file pattern) arranged between collagen fibers [1]. There is a relative sparing of the epidermis in all cases located in the eyelid. The histiocytoid cells appear identical to those in lobular carcinoma of the breast. A proportion of the cells usually have the morphology of signet ring cells with crescent-like nuclei and a main, centrally located cytoplasmic vacuole. On immunohistochemistry, there was androgen receptor stain positivity in our case. Accounting for the case documented by *Sakamoto* et al [5], this is the second case of primary signet-ring cell/histiocytoid carcinoma where androgen receptor stain positivity is reported. In such cases, anti-androgen therapy may have a role in the management of this tumour. Furthermore, GATA3 positivity was noted in our case. Although commonly positive in breast, cutaneous adnexal and urothelial neoplasms, there are no documented cases to our knowledge of GATA3 positivity in cases of primary cutaneous signet-ring cell/histiocytoid carcinoma.

Periorbital metastases from primary infiltrating carcinomas have frequently been reported. Since primary cutaneous signet-ring cell/histiocytoid carcinoma is a diagnosis of exclusion, a thorough systemic workup is important to rule out another primary malignancy before the diagnosis can be established. Notably, the histological features of primary signet-ring cell/histiocytoid carcinoma can be almost indistinguishable from those of signet cell or histiocytoid carcinomas metastatic to the eyelid, especially metastatic lobular breast carcinomas [6–9]. Furthermore, there are reports of primary signet ring cell carcinomas of the gastrointestinal tract [7, 8] and prostatic adenocarcinoma [3] metastasizing to the eyelid.

Treatment modalities for primary cutaneous signet-ring cell/histiocytoid carcinoma include surgery (namely, local excision and/or exenteration) or radiotherapy with or without adjuvant chemotherapy. Our patient initially had wide exenteration, followed by further resection and reconstruction with a free skin muscle graft. Subsequently, he received radiation to the reconstructed site. Up to this point, there is no evidence of recurrence based on post-radiation PET and CT scans. In review of cases by *Tamboon* et al, the recurrence free period ranged from 5 months to 8 years [3]. Of the 33 total documented cases of primary cutaneous signet-ring cell/histiocytoid carcinoma, ten developed metastases over a 10 year follow-up period [1, 3–5, 10–15]. The most common metastatic sites are regional lymph nodes but can also include skin of the head, neck, trunk, respiratory tract, vulva, bone marrow, spine and parotid gland [3]. Only three of the ten recurrent cases were despite non-radical exenteration and adjuvant radiation [4, 14, 15]. Although there is limited data on prognosis after various treatments, exenteration and radiation may be the most definite treatment modality.

There were a number of challenges that were encountered in this case of primary cutaneous signet-ring cell/histiocytoid carcinoma of the eyelid. Firstly, due to its rarity and insidious nature, initial diagnosis and referral was delayed. Primary cutaneous signet-ring cell/histiocytoid carcinoma of the eyelid has a male predominance and often is initially misdiagnosed as blepharoconjunctivitis, chalazion, or other inflammatory disorders. Furthermore, the overlying epidermis is typically spared with no ulceration and most patients are asymptomatic. The clinical course can often be protracted for several years further prolonging the diagnosis. *Tamboon* et al, reported that of all documented cases, the duration between onset and seeking medical attention ranged from 2 months to 7 years. In our case, the patient appeared to have right-sided ptosis for several years as identified in his driver’s license photo. He had noted progressive, right sided ptosis and was referred for surgical ptosis correction. Our case highlights the challenging diagnosis of the primary signet-ring cell/histiocytoid carcinoma due to its prolonged, often subtle presentation.

Secondly, there was difficulty in the interpretation of the intraoperative frozen sections in our case. Due to the lack of desmoplastic response and frozen section artifacts, it is difficult to differentiate tumour cells from inflammatory cells, especially when tumour cells become sparse at the margins. For that reason, intraoperative frozen sections may not be the optimal choice to assess close margins in these cases. Thus, more extensive resections may be required to confirm negative margins. Furthermore, primary cutaneous signet-ring cell/histiocytoid carcinoma can present with numerous subtle microfocal stromal extensions towards the periphery, without much gross clinical disruption. In spite of numerous superficial biopsies of the right periorbital tissue, there were positive margins, highlighting the fact that this tumour may show growth beyond what its clinical appearance suggests.

There are 32 reported cases of primary signet-ring cell/histiocytoid carcinoma in the English literature [1–5, 10–28]. Our case represents the 33th case of primary signet-ring cell/histiocytoid carcinoma of the eyelid in a 73 year old male, and illustrates the diagnostic and treatment challenges of this tumour. Furthermore, this is the first documented case with GATA3 positivity and the second documented case with androgen receptor stain positivity. As such, further research should investigate androgen receptor therapy as a potential future treatment for this rare entity.

## Data Availability

Not applicable.

## Abbreviations

CT: Computed tomography
PET: Positron emission tomography
AE1/AE3: Cytokeratin AE1/AE3
CK7: Cytokeratin 7
GCDFP-15: Gross cystic breast disease fluid protein 15
AR: Androgen receptor
GATA3: GATA binding protein 3
CK20: Cytokeratin 20
CDX2: Protein coding gene (caudal type 2 homeobox)
TTF-1: Thyroid transcription factor 1
S100: S100 protein
PSAP: Prostatic specific acid phosphatase
PAX8: Paired box gene 8
CD20: B-lymphocyte antigen CD20
CD117: Mast/stem cell growth factor receptor
CD43: Leukosialin
WT1: Wilms tumour protein
PSA: Prostate specific antigen
MIB-1: E3 ubiquitin-protein ligase MIB1
